# A Double-Channel Hybrid Deep Neural Network Based on CNN and BiLSTM for Remaining Useful Life Prediction

**DOI:** 10.3390/s20247109

**Published:** 2020-12-11

**Authors:** Chengying Zhao, Xianzhen Huang, Yuxiong Li, Muhammad Yousaf Iqbal

**Affiliations:** 1 School of Mechanical Engineering and Automation, Northeastern University, Shenyang 110819, China; zhaochengying0223@163.com (C.Z.); 13354260226@163.com (Y.L.); 2 Key Laboratory of Vibration and Control of Aero Propulsion Systems Ministry of Education of China, Northeastern University, Shenyang 110819, China; 3 School of Mechanical Engineering, Taiyuan University of Technology, Taiyuan 030024, China; a13840311401@163.com

**Keywords:** RUL prediction, double-channel hybrid model, convolution neural network, bidirectional long short-term memory network

## Abstract

In recent years, prognostic and health management (PHM) has played an important role in industrial engineering. Efficient remaining useful life (RUL) prediction can ensure the development of maintenance strategies and reduce industrial losses. Recently, data-driven based deep learning RUL prediction methods have attracted more attention. The convolution neural network (CNN) is a kind of deep neural network widely used in RUL prediction. It shows great potential for application in RUL prediction. A CNN is used to extract the features of time-series data according to the spatial feature method. This way of processing features without considering the time dimension will affect the prediction accuracy of the model. On the contrary, the commonly used long short-term memory (LSTM) network considers the timing of the data. However, compared with CNN, it lacks spatial data extraction capabilities. This paper proposes a double-channel hybrid prediction model based on the CNN and a bidirectional LSTM network to avoid those drawbacks. The sliding time window is used for data preprocessing, and an improved piece-wise linear function is used for model validating. The prediction model is evaluated using the C-MAPSS dataset provided by NASA. The predicted results show the proposed prediction model to have a better prediction performance compared with other state-of-the-art models.

## 1. Introduction

In recent years, mechanical equipment is becoming more and more complicated, and mechanical reliability is strictly required, so the method of prognostic and health management (PHM) has attracted more and more attention than before [1,2,3,4,5,6]. The prediction of remaining useful life (RUL) is an important part of PHM. Efficient RUL prediction can ensure the development of maintenance strategies and reduce industrial losses. The methods of RUL prediction can be roughly divided into three categories: (1) a physical model approach, (2) data-driven approach, and (3) hybrid method approach.

The physical model approach is to establish a mathematical degradation physical failure model based on the failure mechanism or performance degradation process of the system [7,8]. At present, this method is mainly used in systems with explicit models and requires researchers to have professional knowledge. Moreover, it is challenging to build dynamic mathematical and physical models accurately for multiple failure modes in the system.

The data-driven approach directly uses sensor monitoring data. First, the data are analyzed using signal processing and other technologies. Then, the features reflecting the system degradation and fault are extracted. Subsequently, the system degradation model is established. Finally, the RUL prediction is completed [9,10,11]. The data-driven method is mainly based on experimental data, and then the method generally summarizes the degradation laws to predict RUL. Data-driven methods can be divided into mathematical statistics-based methods and machine learning-based methods. There are many typical mathematical statistical models for RUL prediction, including the autoregressive moving average (ARMA) model [12], Wiener process model [13,14], Markov model [15], etc.

Compared with statistical models, machine learning-based methods have been widely used in recent years. Deep learning is an emerging method in the field of machine learning and has achieved fruitful results in the areas of image processing, speech recognition, and machine translation [16,17,18]. Deep learning can be regarded as a neural network that contains multiple hidden layers. It is an idea that simulates the human brain to learn layer by layer and cause it to realize to learn abstract features from the raw sensor data automatically. It has been widely used in RUL prediction. The neural network can directly establish the relationship between the degradation data of the system and the RUL. Gebraeel et al. [19] proposed an artificial neural network (ANN). Vibration information of bearings from the beginning to the time of failure was collected and was input into the neural network for training. The trained model was used to estimate the failure time of the bearing. Aydemir and Acar [20] proposed an anomaly-triggered RUL estimation method. The data of sensors are monitored, and when statistically significant changes are detected, these are taken as the starting point of degradation to trigger the data-driven RUL prediction model. Fu et al. [21] proposed an adaptive domain autoencoder and long short-term memory (DASE) model to predict the RUL of mechanical parts, which improved the adaptability of the model. Li et al. [22] proposed a neural network with the supervised attention mechanism to screen data with more significant degradation feature information for RUL prediction. Loannis et al. [23] proposed a method to update the RUL prediction of the rolling milling process in real time. The nonparametric statistical process is used to update the prediction. In addition to the above neural network prediction models, models based on self-organizing mapping (SOM) and the backpropagation (BP) neural network model [24], support vector machine (SVM) model [25,26], convolutional neural network (CNN) [27], recurrent neural network (RNN) [28,29], and other models are also applied to RUL prediction. At present, most models cannot fully extract the features of each dimension with multidimensional big data samples. This paper adopts a hybrid model to solve this gap.

The hybrid model method is to integrate the physical model and the data-driven approach. The advantages of the two methods are integrated to improve the prediction performance of the model. Maio et al. [30] combined relevance and exponential regression to realize the RUL prediction. Zheng et al. [31] proposed a hybrid model that combines Kalman filtering and relevance vector machine to predict the degradation trend of batteries. 

As RUL prediction is carried out, the neural network does not need to extract the data feature manually. The neural network has strong feature extraction capability and reduces the problem of overreliance on expert experience and signal processing technology. It is suitable for the requirements of diversity, nonlinearity, and high-dimensional data analysis under the background of big data. When extracting features from time-series data, the CNN is used to extract the features of time-series data according to the spatial feature method. The paper in [32] directly used a deep CNN model to predict the RUL. This way of processing features without considering the time dimension will affect the prediction accuracy of the model. On the contrary, the commonly used LSTM network considers the timing of the data and the interdependence between time-series data. A bidirectional long short-term memory (BiLSTM) network is a combination of two LSTMs, i.e., forward and backward. Based on LSTM, BiLSTM can extract the feature of forward and backward simultaneously. Wang [33] proposed a deep BiLSTM network to predict the RUL. However, compared with CNN, it lacks spatial data extraction capabilities. To solve those gaps and extract deep features from the raw sensor data, this paper proposes a double-channel hybrid prediction model based on CNN and a BiLSTM network. The parallel channels are independent of each other and do not affect each other in the training process, thus improving the prediction accuracy of the model. Based on the C-MAPSS dataset, the model proposed in this paper is compared with the RUL prediction model proposed in recent years, which shows that the double-channel hybrid model has better prediction capability.

The rest of the paper is organized as follows: In Section 2, it introduces the relevant literature review. In Section 3, the CNN and the LSTM network are introduced, and a double-channel hybrid model is proposed. Section 4 presents the data preprocessing method and model evaluation method. Section 5 shows the experimental results, and Section 6 gives the conclusions.

## 2. Literature Review

CNNs have been widely used in RUL prediction due to their excellent property of extracting spatial features from the raw data. Babu et al. [34] proposed a novel regression method based on a deep CNN to estimate RUL. The convolution and pooling filters are applied to multichannel sensor data along the time dimension. The features learned from the raw sensor signal are automatically combined systematically. Through the deep architecture, the model determines the high-level abstract representation of the raw sensor data. Moreover, feature learning and RUL estimation are mutually enhanced through supervised feedback. Ren et al. [35] proposed a method to predict the RUL of a bearing based on a deep CNN. For the first time, a smooth method was innovatively applied to deal with the problem of discontinuous prediction results. Experimental results showed that this method improved the accuracy of the RUL prediction of bearings. A CNN fails to consider the correlation between the time-series of the raw sensor data. For the far-away time data, it is easy to lose its characteristic information, resulting in poor long-term prediction ability and insufficient prediction accuracy. In this paper, bidirectional long short-term memory (BiLSTM) and the CNN are combined to solve this problem effectively.

LSTM networks can capture long-term dependencies between features. They can improve the accuracy of RUL prediction. Zhang et al. [36] used the LSTM neural network to predict the RUL of lithium-ion batteries. The elastic mean square backpropagation method is used to optimize the model. Wu et al. [37] proposed using a vanilla LSTM neural network to obtain better prediction accuracy under complex operation, working conditions, model degradation, and intense noise. Furthermore, a dynamic difference technique was proposed to extract interframe information to enhance the cognitive ability of the model degradation process.

## 3. Methodology

### 3.1. Convolutional Neural Network

**Figure 1:**
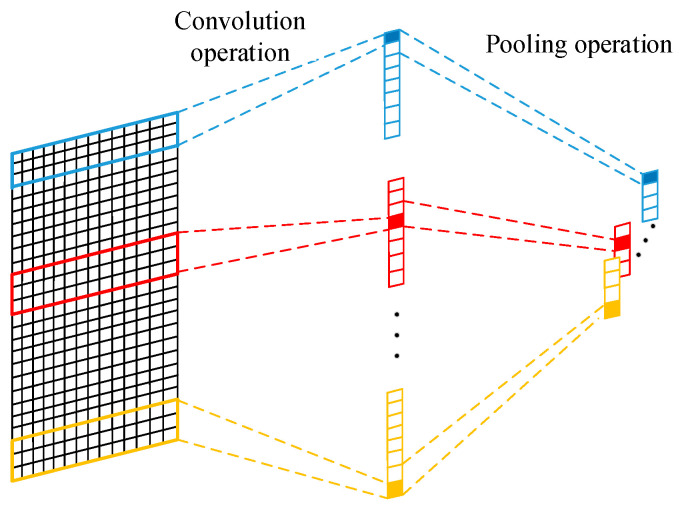
Convolution and pooling operation.

The CNN was firstly proposed by Lecun et al. [38] for image processing. The CNN is a class of feed-forward neural network that contains a convolutional operation and deep structure. Compared with the early backpropagation (BP) neural network, the essential characteristics of CNN are local perception and parameter sharing. It can extract the spatial features from the raw input data and realizing a high-dimensional feature representation of raw data. Figure 1 shows the convolution and pooling operation. The convolutional layer uses convolutional kernels to convolve local regions of the input data to generate corresponding features. The parameter sharing of a CNN decreases the parameters in the calculation process of the neural network. This effectively avoids the phenomenon of having too many parameters, which leads to overfitting the neural network. In this way, the overfitting of the neural network is resolved. Moreover, the system memory occupied in the training process of the neural network significantly decreases. Formula (1) shows the convolution operation of the *i*-th convolution kernel.

(1) $$y_{i}=\varphi \left(W_{i}X_{i-1}+b_{i}\right)$$

where $X_{i-1}$ represents the input of the *i*-th layer CNN; $W_{i}$ represents the *i*-th filter kernel; $b_{i}$ and φ represent the bias term and the nonlinear activation function, respectively.

The final output of one convolution layer is to connect the results of the convolution operation of *n* convolution kernels together, which can be represented as:

(2) $$Z=\left[y_{1},y_{2},\ldots ,y_{n}\right]$$

In this paper, the maximum pooling layer is used to downsample the output of the CNN and extract critical local features. Moreover, the parameters and amount of calculation of the model decreased. Consequently, the operation speed is improved, and the overfitting problem is avoided.

The 2-dimensional convolution network was used in this paper. It is a 1-dimensional operation on the 2-dimensional input data, and it was used to extract the spatial features of the time-series from the input sample.

### 3.2. Bidirectional Long Short-Term Memory Neural Network

**Figure 2:**
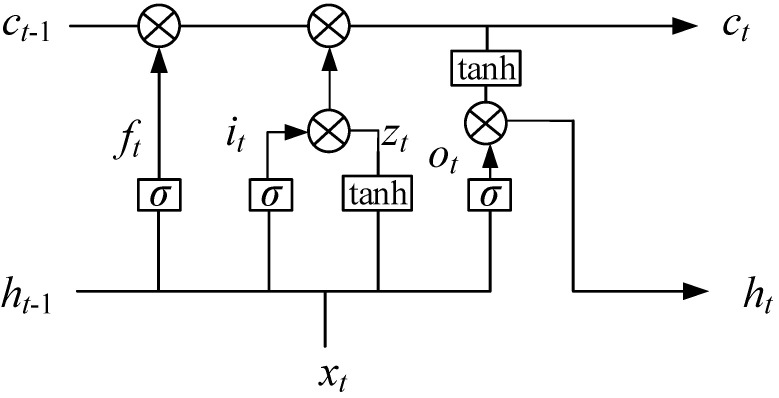
Long short-term memory cell.

In this paper, a BiLSTM was used to extract the long-term dependency characteristics of input sample data. The BiLSTM was composed of two independent LSTM neural networks, and it has two directions of forward and backward propagation. The LSTM network proposed by Hochreiter and Schmidhuber [39] is a unique recurrent neural network (RNN) neural network. It has a particular network structure consisting of an input gate structure and an output gate structure. LSTM affects the state of an RNN at each time through the gate structure. The gates only limit directions of the information flow. The activation function of the gate’s structure is sigmoid. Figure 2 shows the cell of LSTM. 

The core of the LSTM structure is the forget gate and the input gate, which can save long-term memory effectively. The forget gate makes the neural network abandon the previous useless information by the current input $x_{t}$ and the output $h_{t}$ of the last moment. The network overcomes gradient disappearance or gradient explosion during training by importing a gate structure and memory cells. The input gate determines the information to be added to the state $c_{t-1}$ to generate a new state $c_{t}$ according to the parameters $x_{t}$ and $h_{t-1}$. Through the forget gate and input gate structure, the LSTM can effectively determine which information is forgotten and which information is retained. Therefore, LSTM extracts useful information; the output gate determines the output of the $h_{t}$ according to the output $h_{t-1}$ at the moment of the latest state $c_{t}$ and the current input $x_{t}$. The LSTM specific formula is defined as follows:

(3) $$i_{t}=\sigma \left(W_{ix}x_{t}+W_{ih}h_{t-1}+b_{i}\right)$$

(4) $$f_{t}=\sigma \left(W_{fx}x_{t}+W_{fh}h_{t-1}+b_{f}\right)$$

(5) $$o_{t}=\sigma \left(W_{ox}x_{t}+W_{oh}h_{t-1}+b_{o}\right)$$

(6) $$z_{t}=\tan h\left(W_{zx}x_{t}+W_{zh}h_{t-1}+b_{z}\right)$$

(7) $$c_{t}=f\cdot c_{t-1}+i\cdot z$$

(8) $$h_{t}=o\cdot \tan h\left(c_{t}\right)$$

**Figure 3:**
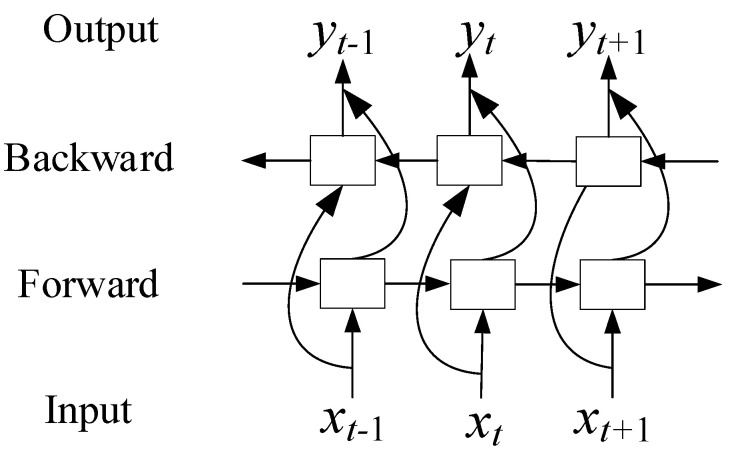
Bidirectional long short-term memory (BiLSTM) neural network.

BiLSTM has two directions in propagation, and both directions are simultaneously transmitted to the output unit. This can capture past and future information, as shown in Figure 3. At each time t, the input is provided to both the forward and backward LSTM networks. The result of the BiLSTM output can be represented as:

(9) $$h_{t}={\vec{h}}_{t}⊙{\overset{\leftarrow }{h}}_{t}$$

where ${\vec{h}}_{t}$ and ${\overset{\leftarrow }{h}}_{t}$ represent the forward and backward results of BiLSTM, respectively.

### 3.3. The CNN-BiLSTM Neural Network Structure

**Figure 4:**
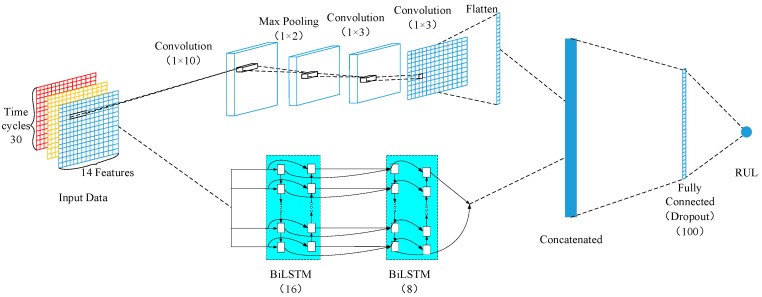
Convolutional neural network (CNN)-BiLSTM hybrid neural network.

In order to fully extract the spatial and temporal features of the input data for RUL prediction, this paper proposes a double-channel hybrid neural network model based on a CNN and a BiLSTM. The structure of the model is shown in Figure 4. The CNN and BiLSTM respectively extract features from the raw data, then concatenate them together and map them to a fully connected layer. The combination of CNN and BiLSTM increases the amount of data, which can then improve the accuracy and stability of prediction.

The input data samples adopt a 2-dimensional structure of $W_{l}\times n$. Here, $W_{l}$ is the length of the time-series, and n is the number of features. In the process of data processing, a sliding time window was used to extract data samples, and the sliding step size of the window is 1. For different datasets of the C-MAPSS, different time-window lengths were used.

The first channel of the hybrid model is composed of three layers of CNN, a layer of maximum pooling, and a layer of flatten. The CNN structure of the first channel was used to extract the spatial features from the sensor’s raw data; however, the depth of time features extraction of the raw high-dimensional data is far from enough. Therefore, the BiLSTM structure was adopted to complete the extraction of the data time-series features and extract the long-term dependencies between data features. Meanwhile, the BiLSTM structure can avoid gradient disappearance and gradient explosion during training the model. The first convolutional layer used 10 filters of size (1 × 10), and the maximum pooling filter of size (1 × 2) is adopted. The second layer of convolution used 10 filters with filter size of (1 × 3). The last layer of the convolutional used a filter of size (1 × 3). If the data dimension is too small, the first channel CNN will fail. In order to prevent failure, the stacked CNN adopted the zero-filling method. The convolutional data were finally input to the flatten layer. The purpose of the flatten layer is to convert the features extracted from the first channel convolution neural network into a 1-dimensional structure, which is convenient for concatenating with the features extracted from the BiLSTM path. The second channel stacked two layers of BiLSTM. Moreover, the cells of the two layers were set to 16 and 8, respectively. The input data were converted to 1-dimensional data before they were inputted into the BiLSTM path.

The feature data matrices extracted from the first channel and the second channel were concatenated, inputting to a fully connected layer containing 100 neurons. To prevent overfitting, the dropout technique was used. The dropout technology stops the hidden layer neurons with self-defined probability numbers from working in the forward propagation of the training process. In doing so, it improves the generalization ability of the model and enhances the robustness of the model. In this paper, the dropout rate is 0.5. Hence, a part of neurons in the network will be hidden with a self-defined probability of 0.5 in each iteration during the training process. A subnetwork will be obtained by randomly sampling the network parameters, and only the subnetwork will be updated in this iteration. All the above layers use “ReLU” as the activation function. Finally, a regression layer containing a neuron was used to predict the target RUL.

The BP algorithm was used to train the model, and the model weights and biases were updated continuously. The Adams optimizer algorithm was used to monitor the training error “MSE” in order to update the model parameters continually. The learning rate was set to 0.001. The mini-batch size was 512, and the maximum training epoch was set as 200.

## 4. Experiments

### 4.1. C-MAPSS Dataset

In this part, the C-MAPSS dataset was used to verify the predictive performance of the proposed double-channel hybrid deep neural network model. The C-MAPSS dataset has four subsets (FD001, FD002, FD003, and FD004), recording 21 different types of sensor data respectively. The monitoring data of all sensors reflect the well-being status of the engine. These four subsets contain different operating conditions and fault modes. Each subset possesses its training dataset and test dataset. The train dataset records the data of all sensors in the whole life cycle from the start of the run to the time of the failure. The test dataset records all sensor data from the start of engine operation to a random cycle point in time. In datasets, the first column shows the serial number of the engine, with the second column representing the number of cycles of the engine, the third to fifth column showing different operating settings, and the sixth to the twenty-sixth column displaying the data recorded by 21 various sensors. The details of the four datasets are shown in Table 1.

### 4.2. Data Preprocessing

The 21 sensors in each subset record different data, and the data range between different sensors varies significantly. The unprocessed data affected the performance of predicting the engine RUL, so it is essential to standardize the data. Data normalization is an essential step in data processing. Additionally, the output value of Sensors 1, 5, 6, 10, 16, 18, and 19 are constant, without providing useful information for RUL prediction. Therefore, the other 14 sensors’ data were selected for normalization processing for RUL prediction.

There are two ways to normalized the data, the Z-score normalization as given in Formula (10), and the min-max standardization as given in Formula (11). Min-max normalization is employed to process the data in this paper.

(10) $$y_{j}^{i}=\left(x_{j}^{i}-{\bar{x}}^{i}\right)/\sigma^{i}$$

(11) $$y_{j}^{i}=\left(x_{j}^{i}-x_{min}^{i}\right)/\left(x_{max}^{i}-x_{min}^{i}\right)$$

where $x_{j}^{i}$ is the *j-*th output of the *i-*th sensor; ${\bar{x}}^{i}$ is the mean value of all outputs of the *i*-th sensor; $x_{min}^{i}$ is the minimum value of all output of the *i-*th sensor; and $x_{max}^{i}$ is the maximum value of all output of the *i-*th sensor.

**Figure 5:**
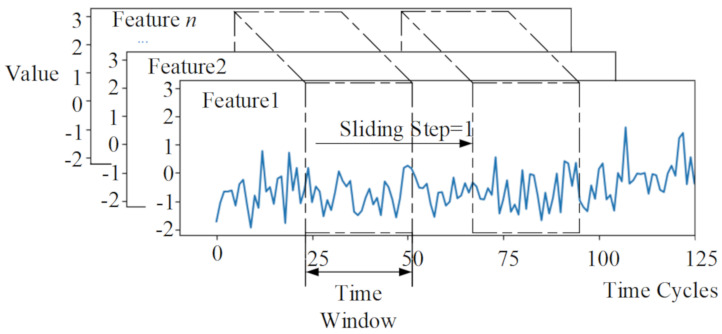
Sliding time window to extract data samples.

In the model of RUL prediction, the cycle number of multivariate time-series included in the input sample data has a direct impact on the prediction performance. The larger the cycle number of samples, the more data information is monitored by sensors, and the better the model’s prediction effect. In this paper, a sliding time window was used to extract samples of standardized data. According to the minimum cycle number of all engines in the four datasets, the time-window lengths of the four datasets are 30, 21, 36, and 18 cycles, respectively, and the sliding step size is one cycle. Figure 5 shows that using a sliding time window to extract input data samples from a multivariate time-series normalized the data. The RUL of the last cycle of the time window was used as the label for the sample. Meanwhile, *n* is the number of engine cycles represented, *l* is the length of the time window, then *n* − *l* + 1 samples can be extracted from the multivariate time-series dataset of the engine.

### 4.3. Evaluation Methods

In this paper, a scoring function and the root mean square error (RMSE) are used to evaluate the performance of the proposed model of RUL estimation. The detailed introduction is shown as follows.

The scoring function is as follows:

(12) $$s_{i}=\{\begin{array}{c} e^{-\frac{h_{i}}{13}}-1\;,\;h_{i}<0\\ e^{\frac{h_{i}}{10}}-1,\;\;\;\;h_{i}>0 \end{array}\;,\;score=\overset{N}{\underset{i=1}{\sum }}s_{i}$$

where *N* is the number of test samples; $h_{i}$ means the error between the $RUL_{\mathrm{prediction}}$ and $\;RUL_{\mathrm{actual}}$; and $h_{i}=RUL_{\mathrm{prediction}}-RUL_{\mathrm{actual}}$. The scoring function was proposed at the International Conference on Prognostics and Health Management Data Challenge (2008). 

The root mean square error (RMSE) is as follows:

(13) $$\mathrm{RMSE}=\sqrt{\frac{1}{N}\overset{N}{\underset{i=1}{\sum }}h_{i}^{2}}$$

**Figure 6:**
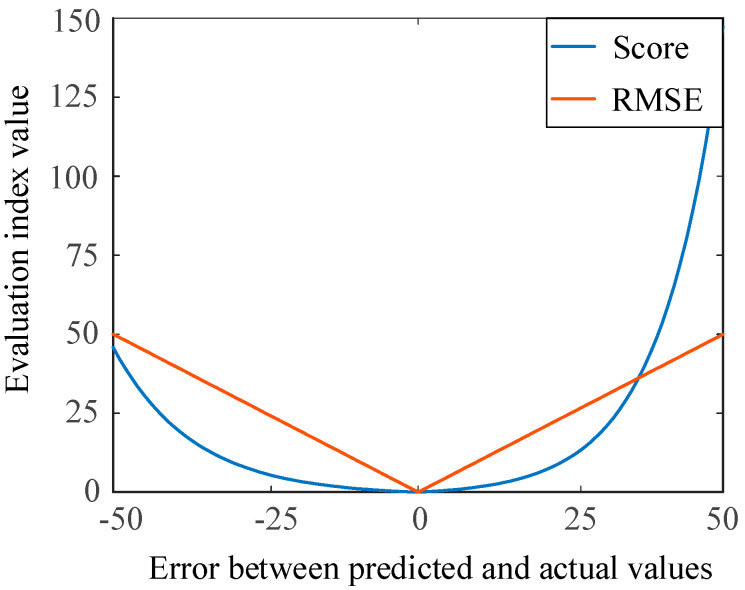
Comparison between two evaluation indicators.

Figure 6 shows two indicators of the evaluation model, i.e., the RMSE and scoring function, giving different penalties for early and late prediction. Value of *h* (*h* = 0) means that the $RUL_{\mathrm{prediction}}$ is equal to $RUL_{\mathrm{actual}}$, and the two indicators receive a zero. When the error increases, the values of the RMSE and the score also increase correspondingly. RMSE can directly reflect the overall RUL prediction error and is also one of the commonly used indexes. For the scoring function, if the predicted value is less than the actual value, it is predicted early, and the penalty coefficient is small. In contrast, late prediction induced a large penalty coefficient. The advance prediction of the RUL can make maintenance plans and reduce losses in industries. RMSE has the same penalty for early and late prediction.

### 4.4. RUL Target Function

**Figure 7:**
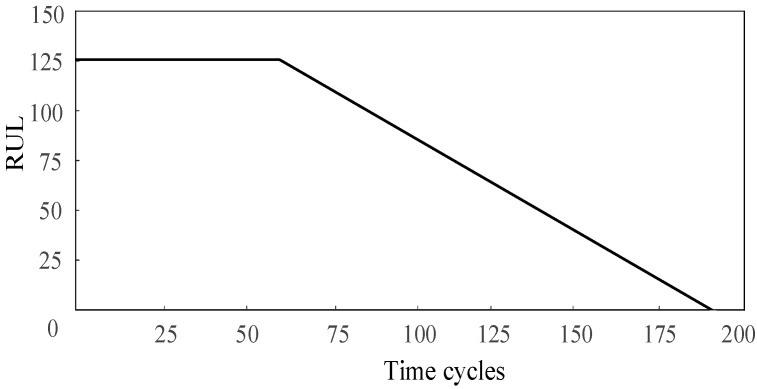
The piece-wise linear remaining useful life (RUL) target function.

The RUL in the C-MAPSS dataset was considered to decrease linearly with time when predicting the RUL. In practice, the performance of the machine is stable at the beginning of work, and the degradation of the machine can be ignored. However, the RUL of mechanical parts decreases linearly with time due to various working conditions such as wear and vibration. A piece-wise linear RUL method is presented in [29]. Therefore, this study also used a piece-wise linear function to represent the RUL, as shown in Figure 7, and the maximum RUL was set to 125.

## 5. Results and Analysis

**Figure 8:**
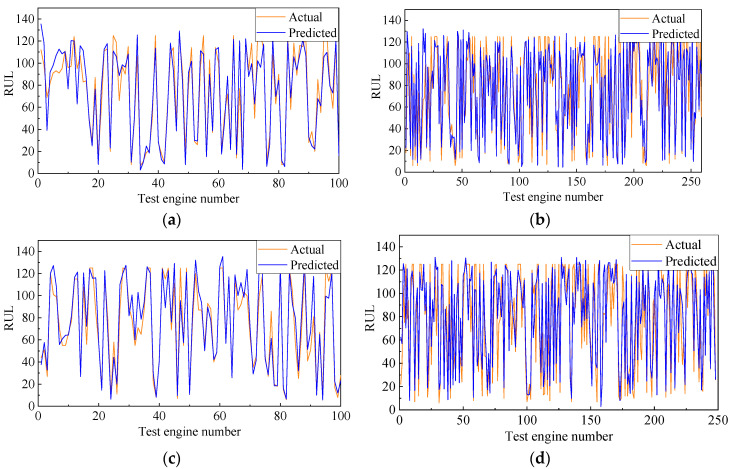
Predicted and actual values of (**a**) FD001 dataset, (**b**) FD002 dataset, (**c**) FD003 dataset, and (**d**) FD004 dataset.

**Figure 9:**
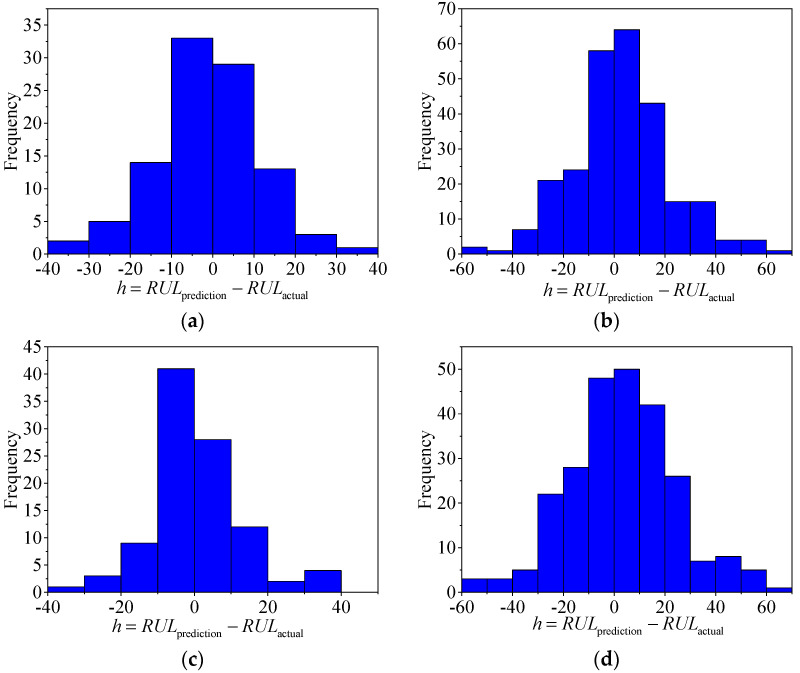
The frequency distribution of the prediction error of (**a**) FD001 dataset, (**b**) FD002 dataset, (**c**) FD003 dataset, and (**d**) FD004 dataset.

In this section, the double-channel hybrid prediction model based on the CNN and BiLSTM is evaluated by the C-MAPSS datasets. For the four datasets of the C-MAPSS, a different sliding time-window length is used to process the data samples, and then we input the model for training. The model is evaluated by the RMSE and scoring function. The last time-window data of all engines in the four test datasets are selected to predict the RUL. The prediction results are shown in Figure 8. The solid black line indicates the actual RUL of the engine, and the solid red line represents the predicted RUL. The predicted values of the proposed model are very close to the actual values. Figure 9 shows the error frequency distribution between the predicted and actual RUL of the engine for the C-MAPSS datasets.

In previous work [32,34,40,41], different deep learning models were established, and predictable the performance through the C-MAPSS dataset was analyzed. Table 2 and Table 3 show the RMSE and score comparison between the proposed hybrid network model and other reported models. The result indicated that the proposed model shows a smaller RMSE and score in the four datasets. The improved ratio is the degree to which the RMSE/score is improved compared with the other papers’ models. Compared with other models, the apparent improvement of the score index shows that the hybrid network model can better realize RUL prediction ahead of time, thus realizing predictive maintenance of industrial production. The significant improvement of the RMSE index indicates that the model has a more accurate prediction ability.

The prediction capability of the model proposed in this paper is significantly improved. Compared with the traditional MLP, SVR, and RVR models, the proposed model does not need artificial feature extraction. It can realize independent feature extraction in the time dimension and space dimension, with better applicability, smaller error, and better prediction performance. Compared with CNN, LSTM, and BiLSTM networks, the double-channel neural network extracts features independently of each other and then combines the features extracted by the two channels, making full use of the advantages of the CNN and BiLSTM networks, increasing the amount of data and improving the prediction accuracy.

The prediction results of the FD001 and FD003 datasets with smaller errors are dramatically better than the FD002 and FD004 datasets. Because the operating conditions of the FD002 and FD004 datasets are more complicated than other datasets, the operation mode and failure mode affected the working process of the system and increased the difficulty of prediction. The FD002 and FD004 datasets contain six different operating conditions. In contrast, FD001 and FD003 have only one operating condition. The scores predicted by the FD002 and FD004 datasets are large, caused by a large number of engines included in these two datasets.

**Figure 10:**
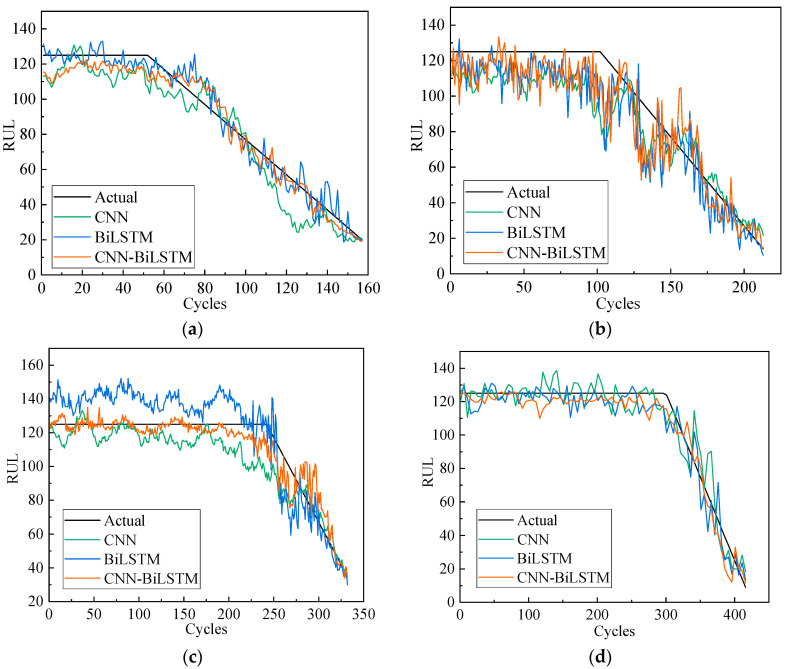
Different models predicting the whole life cycle of 4 engines with their predicted RUL and the actual RUL: (**a**) Unit #24 in FD001, (**b**) Unit #64 in FD002, (**c**) Unit #71 in FD003, (**d**) Unit #135 in FD004.

Figure 10 shows the comparison of the predicted and actual RUL values for the four engines selected from the test datasets of FD001, FD002, FD003, and FD004 with the double-channel hybrid CNN-BiLSTM model, single-channel CNN model, and single-channel BiLSTM model. The 135th engine data in the FD004 data set has 435 cycles. In order to facilitate observation, a set of data is drawn from every four points in this paper. It can be seen from the figure that compared with the CNN and BiLSTM model, the prediction effect of most engines using the double-channel CNN-BiLSTM model in this paper is better than other methods. Moreover, the predicted RUL fluctuates around a constant value in the early periods. With the increase in the number of engine cycles, the actual and predicted RUL values decrease linearly in line with the reality of the engine degradation phenomenon. However, the expected value is close to the real value in the last few cycles. With the increase of engine cycles, the model’s ability to capture engine degradation is significantly improved. In the latter stage, the model’s predictive ability becomes even better. The model proposed in this paper shows good predictive ability.

**Figure 11:**
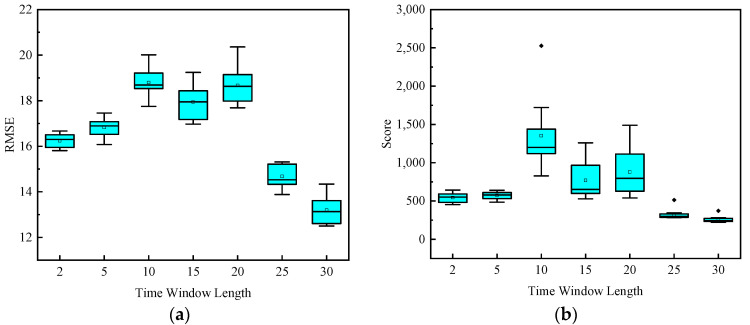
Box diagram of the evaluation index for the influence of the time-window length on model prediction performance: (**a**) RMSE (**b**) Score

Figure 11 shows the effect of the sliding time-window length on the model’s predictive performance when extracting samples in the FD001 dataset. In this paper, the length of 7 time-windows with different sizes was selected from the FD001 dataset, i.e., 2, 5, 10, 15, 20, 25, and 30. Other parameters of the model were set in the same way, and the box plot drawn by the RMSE and score was obtained after ten runs. Figure 10 shows the better performance of the model after training the data samples extracted with a large time-window length. In a certain range, the larger the window length, the more information contained in the extracted samples, which is more conducive to the extraction of data features by the model. The minimum cycle number of the engine in the dataset in the FD001 dataset is 31 cycles, and the time-window length is 30 cycles.

Table 4 shows the performance comparison between the only CNN channel model, the only BiLSTM channel model, and the hybrid model on the FD001 dataset. The only CNN channel model is mainly composed of three convolution layers, a maxpooling layer, a fully-connection layer, and a regression layer. The only BiLSTM channel model is mainly composed of two BiLSTM network layers, a fully-connection layer and a regression layer. All parameter settings are consistent with those of the hybrid model proposed in this paper. It was observed that the performance of the proposed hybrid model is significantly better than that of the other two models. The hybrid model combines the advantages of the CNN and BiLSTM to extract the temporal and spatial features of the raw sensor data; the fully-connection layer increases the output of the nonlinearity. Finally, the regression layer was used to implement the RUL prediction.

## 6. Conclusions

This paper proposed a double-channel hybrid prediction model based on a CNN and BiLSTM network. The sliding time-window was used for data preprocessing, and an improved piece-wise linear function was used for model validating. The prediction model was evaluated using the C-MAPSS dataset provided by NASA. This paper can be concluded as follows:

1. Compared with other state-of-the-art models, the hybrid model gets a better RMSE and score. The results show that the proposed double-channel hybrid model has a better predictive capability. The data of four engines in the dataset were extracted for prediction. The model can predict the degradation trend of engine performance.
2. Within a specific time-window length range, increasing the length of the time window will improve the RUL prediction accuracy of the double-channel hybrid model.
3. Compared with the prediction performance of the CNN channel model and the BiLSTM channel model proposed in this paper, the hybrid model exhibits better prediction capability.

## Figures and Tables

**Table 1:** Introduction of the C-MAPSS dataset.

Dataset	C-MAPSS			
	FD001	FD002	FD003	FD004
Train trajectories	100	260	100	249
Test trajectories	100	259	100	248
Operating conditions	1	6	1	6
Fault modes	1	1	2	2
Training samples	17731	48819	2Tab820	57522
Testing samples	100	259	100	248

**Table 2:** RMSE comparison based on C-MAPSS dataset.

Method	C-MAPSS			
	FD001	FD002	FD003	FD004
MLP [34]	37.56	80.03	37.39	77.37
SVR [34]	20.96	42.00	21.05	45.35
RVR [34]	23.80	31.30	22.34	34.34
CNN [34]	18.45	30.29	19.82	29.16
MODBNE [40]	15.04	25.05	12.51	58.66
LSTM [41]	16.14	24.49	16.18	28.17
BiLSTM [33]	13.65	23.18	13.74	24.86
DCNN [32]	12.61	22.36	12.64	23.31
Proposed method	12.58	19.34	12.18	20.03
Improved ratio	2.38%	13.51%	3.64%	14.07%

**Table 3:** Score comparison based on C-MAPSS dataset.

Method	C-MAPSS			
	FD001	FD002	FD003	FD004
MLP [34]	1.80 × 10^4^	7.80 × 10^6^	1.74 × 10^4^	5.62 × 10^6^
SVR [34]	1.38 × 10^3^	5.90 × 10^5^	1.60 × 10^3^	3.71 × 10^5^
RVR [34]	1.50 × 10^3^	1.74 × 10^4^	1.43 × 10^3^	2.65 × 10^6^
CNN [34]	1.29 × 10^3^	1.36 × 10^4^	1.6 × 10^3^	7.89 × 10^3^
MODBNE [40]	3.34 × 10^2^	5.59 × 10^3^	4.21 × 10^2^	6.56 × 10^3^
LSTM [41]	3.38 × 10^2^	4.45 × 10^3^	8.52 × 10^2^	5.55 × 10^3^
BiLSTM [33]	2.95 × 10^2^	4.13 × 10^3^	3.17 × 10^2^	5.43 × 10^3^
DCNN [32]	2.74 × 10^2^	1.04 × 10^4^	2.84 × 10^2^	1.25 × 10^4^
Proposed method	2.31 × 10^2^	2.65 × 10^3^	2.57 × 10^2^	3.40 × 10^3^
Improved ratio	15.69%	35.84%	9.51%	38.74%

**Table 4:** Performance comparison between single channel model and hybrid model.

Method	RMSE	Score
Only CNN channel	15.32	318
Only BiLSTM channel	18.86	793
Hybrid model	12.51	224
